# Overweight and prognosis in triple-negative breast cancer patients: a systematic review and meta-analysis

**DOI:** 10.1038/s41523-021-00325-6

**Published:** 2021-09-10

**Authors:** Sixten Harborg, Robert Zachariae, Julia Olsen, Maja Johannsen, Deirdre Cronin-Fenton, Henrik Bøggild, Signe Borgquist

**Affiliations:** 1 Department of Oncology, Aarhus University Hospital/Aarhus University, Aarhus, Denmark; 2 Department of Clinical Epidemiology, Aarhus University, Aarhus, Denmark; 3 Department of Psychology and Behavioural Sciences, Aarhus University, Aarhus, Denmark; 4 Public Health and Epidemiology Group, Department of Health Science and Technology, Aalborg University, Aalborg, Denmark; 5 Department of Oncology and Pathology, Clinical Sciences, Lund University, Lund, Sweden

**Keywords:** Breast cancer, Cancer epidemiology, Outcomes research

## Abstract

We conducted a systematic review and meta-analysis investigating the association between overweight and outcome in triple-negative breast cancer (TNBC) patients. We searched PubMed and Embase using variations of the search terms *triple-negative breast cancer (population), overweight and/or obesity (exposure), and prognosis (outcome)*. Based on the World Health Organization guidelines for defining overweight, we included longitudinal observational studies, which utilized survival statistics with hazard ratios (HRs) in our analysis. The included studies measured body mass index at the time of diagnosis of TNBC and reported disease-free survival and/or overall survival. Study quality was assessed with the Newcastle-Ottawa Scale and study data were extracted using the Meta-analysis of Observational Studies in Epidemiology (MOOSE) checklist, independently by two authors. Random-effects models were used to combine the effect sizes (HRs), and the results were evaluated and adjusted for possible publication bias. Thirteen studies of 8,944 TNBC patients were included. The meta-analysis showed that overweight was associated with both shorter disease-free survival (HR = 1.26; 95%CI: 1.09–1.46) and shorter overall survival (HR = 1.29; 95%CI: 1.11c1.51) compared to normal-weight. Additionally, our Bayesian meta-analyses suggest that overweight individuals are 7.4 and 9.9 times more likely to have shorter disease-free survival and overall survival, respectively. In conclusion, the available data suggest that overweight is associated with shorter disease-free and overall survival among TNBC patients. The results should be interpreted with caution due to possible publication bias.

## Introduction

Every year, around 2.1 million women are diagnosed with breast cancer worldwide^1^. Alongside increasing breast cancer incidence; overweight and obesity have become growing health issues^2^. The World Health Organization (WHO) reports that approximately 40% of the world’s female population is overweight with a body mass index (BMI) of 25 kg/m^2^ or above, and 15% are obese (BMI 30 kg/m^2^ or above)^3^. These numbers continue to increase^3^.

Not only is overweight a risk factor for developing breast cancer^4^, but it is also associated with a less favorable breast cancer prognosis^5^, with higher BMI having been found associated with increased risk of recurrence and mortality of breast cancer, irrespective of hormone-receptor (HR) status^5,6^. While BMI may not be an optimal indicator of body composition,^7^ BMI remains the current standard tool for measuring and defining overweight and obesity^8^.

While the majority of breast cancers are HR-positive, globally around 10% are triple-negative breast cancers (TNBC)^9^. TNBC is characterized by cancer progression independent of estrogen, progesterone, and human epidermal growth factor 2 protein (HER2), and is 10–20% more common in overweight compared with normal-weight women^10,11^.

There are several plausible biological reasons for a negative prognostic role of overweight in TNBC. First, in an obesity setting, released cytokines shift from an anti-inflammatory to a pro-inflammatory/proangiogenic profile^12^. Second, in obesity, circulating chemokine ligand 2 (CCL2) levels are elevated^13^. High CCL2 levels are associated with increased presence of tumor-associated macrophages^14^, which can change their phenotypes depending on the tumor microenvironment and promote tumor growth and progression^15^. Finally, free fatty acids produced by adipose tissue lipolysis in obese individuals stimulate toll-like receptor 4 on breast cancer cells and induce activation of the nuclear factor – kappa B pathway (NF-κB)^16^, with continuous NF-κB activation leading to an increase in cancer stem cells in vitro^17^.

Compared with HR-positive breast cancer, TNBC is associated with increased risk of developing metastatic disease and lower survival rates^18^. Chemotherapy is the systemic treatment of choice in the neoadjuvant or adjuvant setting for TNBC patients. Although patients with TNBC have an increased likelihood of pathologic complete response when treated with neoadjuvant chemotherapy compared to breast cancer patients with other subtypes^19,20^, no targeted therapy is yet available for TNBC patients^18^.

So far, only one published meta-analysis has explored the association between overweight and TNBC prognosis^21^. The meta-analysis by Mei et al.^21^ is limited by being restricted to studies of obese TNBC patients, rather than both overweight and obese patients, and by using another measure than the hazard ratio, i.e., the summary odds ratio, as the clinical outcome effect size.

Given the poor prognosis of TNBC and the global obesity epidemic^2^, we evaluated the association of overweight, a potentially modifiable lifestyle-related factor, with the prognosis of TNBC by conducting a systematic review and meta-analysis of the available evidence.

## Results

### Study characteristics

**Fig. 1:**
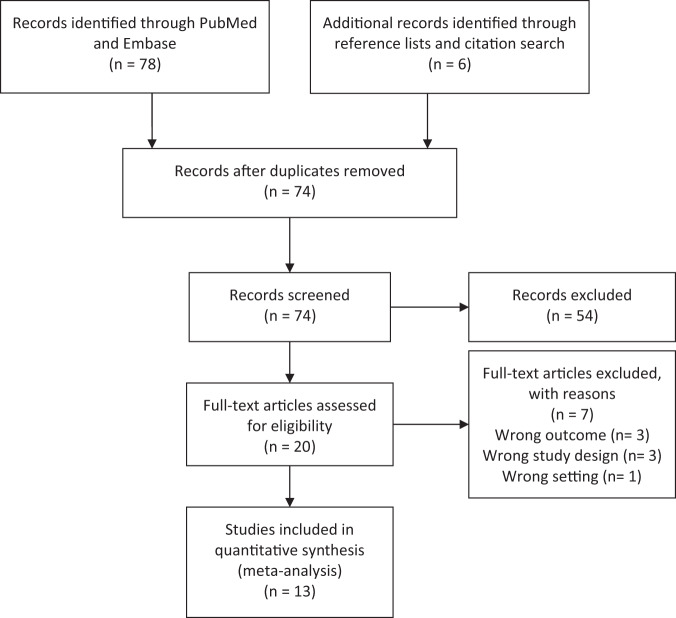
PRISMA Flow Diagram of selected studies. * PRSIMA = Preferred Reporting Items for Systematic Reviews and Meta-Analyses.

The search and subsequent study selection process resulted in the inclusion of 13 independent studies investigating a total of 8,944 TNBC patients. Of these, ten studies of 5,109 patients reported data on DFS^22–31^, twelve studies of 8,005 patients reported OS data^22–24,26–34^, and nine studies reported both endpoints^22–24,26–29^. The study selection process, including reasons for exclusion, is shown in Fig. 1, and the study characteristics are listed in Table 1. The median follow-up time ranged from 24 to 109 months, with an average of 54.9 months across studies.

**Table 1:** Study characteristics of included studies.

Study (year published)	Country	Study period	BMI comparator groups	Population	Menopausal status	Ascertainment of TNBC status	Median follow-up months (years)	HR for shorter disease-free survival	HR for shorter overall survival
*Ademuyiwa (2011)*^47^	*United States*	*1996*–*2010*	(<25) vs (≥25)	418	N/R	*Immunohistchemical analyses by investigators*	37.2 (3.1)	N/R	0.94 (0.61–1.42)
*Dawood (2012)*^44^	*United States*	*1990*–*2010*	(<25) vs (≥25)	2311	*Pre-and postmenopausal*	*Immunohistchemical analyses by investigators*	39 (3.3)	N/R	0.99 (0.83–1.18)
*Sparano (2012)*^43^	*United States*	*1989*–*2002*	(<25) vs (≥30)	878	*Pre-and postmenopausal*	*Biochemical assay or positive immunohistochemistry according to individual institutional standards*	95 (7.9)	1.02 (0.8–1.3)	1.11 (0.85–1.46)
*Pajares (2013)*^44^	*Spain*	*1996*–*2008*	(<25) vs (≥35)	973	*Pre-and postmenopausal*	*Immunohistchemical analyses by investigators*	93.4 (7.8)	1.4 (0.9–2.3)	1.4 (0.9–2.2)
*Mowad (2013)*^36^	*United States*	*1998*–*2011*	(<25) vs (≥25)	183	*N/R*	*Extracted from pathology reports*	42.5 (3.5)	1.01 (0.67–1.52)	1.36 (0.77–2.42)
*Turkoz (2013)*^42^	*Turkey*	*2001*–*2011*	(<25) vs (≥30)	107	*Premenopausal*	*Extracted from pathology reports*	29 (2.4)	1.4 (1.0–2.0)	1.4 (1.0–2.1)
*Tait (2014)*^40^	*United States*	*2006*–*2010*	(<25) vs (≥25)	448	*Pre-and postmenopausal*	*Extracted from pathology reports*	40.1 (3.3)	1.01 (0.65–1.56)	1.22 (0.78–1.91)
*Widschwendter (2015)*^35^	*Germany*	*2005*–*2007*	(<25) vs (≥25)	742	*Pre-and postmenopausal*	*Extracted from pathology reports*	65 (5.4)	1.40 (0.83–2.39)	1.54 (0.85–2.78)
*Hao (2015)*^45^	*China*	*2002*–*2012*	(<24) vs (≥24)	1106	*Pre-and postmenopausal*	*Immunohistchemical analyses by investigators*	44.8 (3.7)	N/R	1.46 (1.04–2.06)
*Chen (2016)*^37^	*China*	*2006*–*2015*	(<25) vs (≥25)	206	*Pre-and postmenopausal*	*Immunohistchemical analyses by investigators*	59 (4.9)	1.55 (0.89–2.73)	1.9 (0.98–3.71)
*Bao (2016)*^41^	*China*	*2002*–*2006*	(<24) vs (≥24)	518	*Pre-and postmenopausal*	*Extracted from medical charts and receptor assays*	109 (9.1)	1.38 (0.88–2.17)	1.19 (0.79–1.81)
*Al Jarroudi (2017)*^39^	*Morocco*	*2009*–*2011*	(<25) vs (≥25)	115	*Pre-and postmenopausal*	*Extracted from pathology reports*	36 (3)	1.89 (1.05–3.43)	2.90 (1.55–5.43)
*Wang (2019)*^38^	*China*	*2005-2015*	(<25) vs (≥25)	939	*Pre-and postmenopausal*	*Immunohistchemical analyses by investigators*	24 (2)	2.33 (1.06–5.12)	N/R

*HR* hazard ratio, *CI* confidence interval, *N/R* Not reported.

### Risk of bias

The risk of bias assessment for each study is shown in Table 4. No study was considered to have a “high risk” of bias. All studies failed to demonstrate that the incident breast cancer for inclusion in their observational analyses was not a recurrent cancer. Furthermore, three studies did not adjust for age and four did not adjust for chemotherapy in their statistical analyses.

### Association between overweight and disease-free survival

**Fig. 2:**
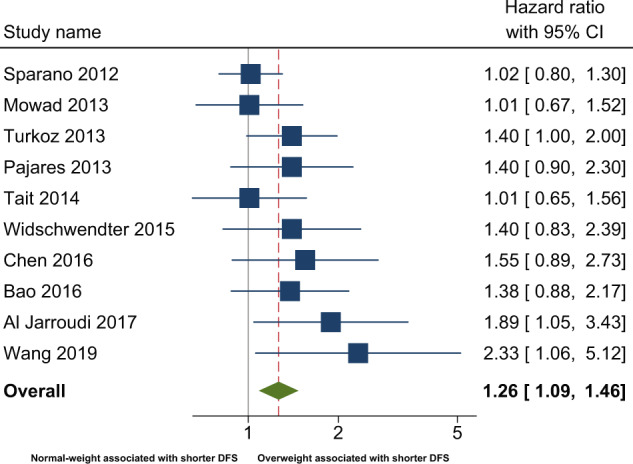
Meta-analysis of observational studies comparing disease-free survival in overweight and normal-weight. * Disease-free survival was defined as time from breast cancer diagnosis to first breast cancer event or death.

**Fig. 3:**
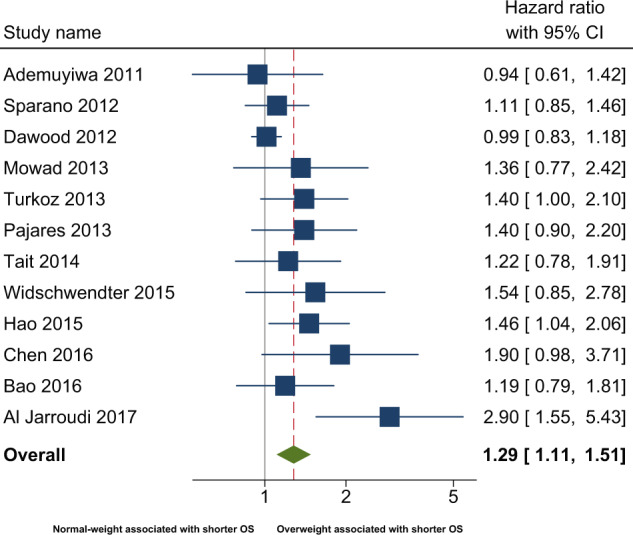
Meta-analysis of observational studies comparing overall survival in overweight and normal-weight. * Overall survival was defined as time from breast cancer diagnosis to death of any cause.

In the primary analysis, the overall combined hazard ratio for DFS indicated that overweight was associated with shorter DFS (HR: 1.26 [95% CI: 1.09–1.46]) (Fig. 2 and Table 2). The non-significant (*p* = 0.31) Q-statistic and the *I*^2^ of 17.74 suggested limited heterogeneity. Egger’s test was statistical significant (*p* = 0.01), and the adjustment based on the five imputed “missing studies” suggested by the trim and fill procedure (Fig. 3A), resulted in a smaller (1.13) hazard ratio, which no longer reached statistical significance. Sensitivity analyses after excluding studies not defining DFS as time from breast cancer diagnosis to first recurrence^22,25,27^ yielded similar results (HR: 1.30 [95% CI: 1.05–1.60]) (Fig. 1). Further, analyses excluding studies with a BMI cut-off of 24 kg/m^2^ did not attenuate the association (HR: 1.33 [95% CI: 1.10–1.61]). When exploring the association between DFS in BMI subgroups differing overweight (defined as BMI 25–30) and obese (defined as BMI≥30) patients from each other, the association was attenuated (Table 3).

**Table 2:** Pooled effects of overweight on disease-free survival and overall survival.

BMI comparison group		Sample size		Heterogeneity			Pooled effect			
	Dependent variable	K^a^	N	Q^b^	*p*	*I*^2^	HR^c^	95% CI	*p*	95% PI^d^
BMI ≥ 25 vs BMI < 25	Disease-free survival	10	5,109	10.5	0.31	14.0	1.26	1.09–1.46	<0.01	N/A
	*Adjusted for publication bias*^*e*^	*15*	—	—	—	—	*1.13*	*0.97*–*1.32*	*Ns*	—
BMI ≥ 25 vs BMI < 25	Overall survival	12	8,005	20.4	0.04	46.0	1.29	1.11–1.51	<0.01	0.83–2.34
	*Adjusted for publication bias*^*e*^	*18*	—	—	—	—	*1.08*	*0.92*–*1.27*	*Ns*	—

^a^K = number of studies.

^b^Q-statistic: *p*-values < 0.1 and I^2^ > 0.0 taken to suggest heterogeneity.

^c^HR = hazard ratio (random effects), HR > 1.0 indicate overweight associated with poorer prognosis/increased mortality.

^d^95% prediction interval calculated for heterogeneous results.

^e^As results (Egger’s test and funnel plot) suggested publication bias, missing studies were imputed and the pooled HR adjusted accordingly with the Duval and Tweedie Trim and Fill test^31^, *K* = number of published studies + number of imputed studies.

N= number of patients, Ns = Not significant, N/A = Not applicable.

**Table 3:** Pooled effects of body mass index subgroups on disease-free survival and overall survival.

BMI comparison group		Sample size		Heterogeneity			Pooled effect			
	Dependent variable	K^a^	N^b^	Q^c^	*p*	*I*^2^	HR^d^	95%CI	*p*	95%PI^e^
BMI 25-30 vs BMI < 25	Disease-free survival	3	1,708	1.2	0.55	0.0	1.24	0.97–1.58	0.09	N/A^f^
BMI 25-30 vs BMI < 25	Overall survival	5	4,437	5.6	0.24	27.9	1.07	0.88–1.31	0.52	0.65–1.77
BMI ≥ 30 vs BMI < 25	Disease-free survival	6	3,666	5.05	0.41	1.0	1.14	0.98–1.33	0.08	N/A
BMI ≥ 30 vs BMI < 25	Overall survival	8	6,395	6.2	0.51	0.0	1.09	0.97–1.22	0.15	N/A

^a^K = number of studies, ^*b*^N = number of patients.

^c^Q-statistic: *p*-values < 0.1 and I^2^ > 0.0 taken to suggest heterogeneity.

^d^HR = hazard ratio (random effects), HR > 1.0 indicate overweight associated with poorer prognosis/increased mortality.

^e^95% prediction interval calculated for heterogeneous results.

^f^N/A = Not applicable.

The findings were supported by the supplementary Bayesian Model-Averaged meta-analysis, which provided moderate evidence for a non-zero effect of overweight on DFS corresponding to a Bayes Factor (BF)^35^ of 7.4, i.e., indicating that the alternative hypothesis is 7.4 times more likely than the null-hypothesis. In contrast, the Bayesian analysis provided only weak evidence concerning heterogeneity of the effects. The BF for heterogeneity of 0.64 indicated that the probability that the effect sizes are heterogeneous is only half the probability that they are not heterogeneous. The combined effect size found in the Bayesian meta-analysis was 1.25, which is similar to the effect found with the frequentist approach (1.26). The credible interval, i.e., the interval that the true effect sizes are assumed to lie within with 95% probability was 1.08 to 1.45 and similar to the confidence interval (1.09–1.46).

### Association between overweight and overall survival

In the primary analysis, overweight TNBC patients had a shorter overall survival (OS) in comparison with normal-weight patients (HR: 1.29 [95% CI: 1.11–1.51]) (Fig. 2B and Table 2). The significant Q-statistic and *I*^2^ of 46% suggested that a little less than half of the variation in hazard ratios could be explained by true differences between studies. The subsequently calculated prediction interval suggested that the hazard ratios of 95% of future studies from the same family of studies will lie within a broad interval from 0.83 to 2.34. The funnel plot (Fig. 3b) and Egger’s test (*p* = 0.005) suggested the possibility of publication bias in favor of larger hazard ratios, and imputing six “missing studies” resulted in a reduction of the combined hazard ratio from 1.28 to 1.08, which no longer reached statistical significance. When attempting to explore possible sources of heterogeneity with meta-regression-based moderator analyses, the strength of the association between overweight and OS was not modified by between-study differences in either BMI cut-offs or median follow-up time. A sensitivity analysis excluding studies with a BMI cut-off of 24 kg/m^2^, strengthened the association (HR: 1.46 [95% CI: 1.13–1.89]). When analyzing the smaller number of studies, which provided data on BMI subgroups, i.e., overweight (defined as BMI 25–30) and obese (defined as BMI ≥ 30) patients, separately, the associations with OS did not reach statistical significance (Table 3).

The supplementary Bayesian model provided strong support^35^, i.e., a BF = 9.94, for a non-zero effect of overweight on OS. Furthermore, the BF of 2.97 indicated moderate evidence for a random model, i.e., for heterogeneous effects. The combined effect size found in the Bayesian meta-analysis was 1.23, which is only slightly smaller than the effect found with the frequentist approach (1.29), and the credible interval was 1.07 to 1.47 and similar to the confidence interval (1.11-1.51).

## Discussion

Taken together, the results of the present systematic review and meta-analysis support the hypothesis that overweight TNBC patients have a poorer prognosis with shorter disease-free and overall survival in comparison with normal-weight TNBC patients. During the follow-up periods, overweight TNBC patients were found to be 29% more likely to die than normal-weight TNBC patients.

Bayesian meta-analysis provided additional support^35^ for both findings by indicating that a non-zero effect of overweight was 7.4 times more likely than the null-hypothesis for DFS and approximately 10 times more likely for OS. In addition, heterogeneity was moderate for both endpoints and the risk of bias of the included studies was generally low. While the available data support a conclusion that overweight is associated with poorer prognosis in breast cancer patients with triple-negative disease, the possibility of publication bias in favor of stronger associations between overweight and poorer survival should be taken into consideration when interpreting the results.

While our study thus has several strengths, some limitations should also be noted. First, only few studies have yet explored the prognostic role of overweight in TNBC and the sample sizes are limited by the relatively low prevalence of TNBC. Second, the heterogeneity of the chemotherapy regimens used across the available studies may limit the interpretability of the results. Third, chemotherapy being the current standard systemic treatment of TNBC, is dosed based on body surface area to achieve the optimal pharmacologic biological availability^36^, and BMI is usually strongly associated with body surface area^37^. Thus, it cannot be excluded that the reported negative influence of overweight on TNBC prognosis could at least be partly due to differences in drug dosage determination guidelines^38^, as overweight patients may receive capped doses of chemotherapy to reduce the risk of toxicities^39^. Fourth, in the quality assessment of the included studies, no study was able to verify that the breast cancer diagnosis was not a recurrent breast cancer. Therefore, we cannot exclude the possibility of a classification bias, i.e., classifying a breast cancer diagnosis as the primary cancer despite the fact that it is second cancer. However, given the frequency of second/third cancers it is unlikely that the observed association can be explained by this. Finally, the present review could possibly be limited by two of the included studies^28,33^ using a lower BMI cut-off for overweight (24 kg/m^2^ or above), which could theoretically induce a classification bias^40^. However, these BMI cut-offs were based on WHO standards for the geographic area of the studies, thereby reflecting the population characteristics^41^.

In a recent phase II trial of genomically directed therapy after neoadjuvant chemotherapy in TNBC patients by Ballinger et al., no association between overweight or obesity and clinical outcome was observed^42^. Unfortunately, the estimates presented in the study by Ballinger et al., do not include information on the precision of the estimates, which was a requirement for inclusion in our meta-analysis. The study by Ballinger et al. is important, as it is restricted to chemoresistant TNBC patients and highlights that chemoresistant TNBC patients might constitute a group of patients in which lifestyle interventions lack a beneficial effect. However, this needs to be further addressed in future studies and the results from Ballinger et al., should be interpreted with caution as they are limited by the small population of 172 chemoresistant TNBC patients with residual disease and the short follow-up time (primary endpoint is two year DFS). Furthermore, in a recent meta-analysis evaluating all breast cancer subtypes, obesity (BMI ≥ 30 kg/m^2^), however not overweight (BMI 25-30 kg/m^2^), was reported to influence prognosis in TNBC patients^43^. Compared to this meta-analysis, the partly different results by Lohmann, et al., may be explained by their inclusion of observational studies with heterogeneous BMI comparator groups, heterogeneous endpoints and interventional cohorts. Nonetheless, the limited power in both meta-analyses, calls for further studies investigating the association.

A number of additional issues need to be addressed in future research. The present meta-analysis included studies performed in developing countries^26,29^, and the results thus represent very diverse health care systems and differences in socioeconomic status of patients. Socioeconomic status is generally associated with overweight and women with lower socioeconomic status tend to have lower screening attendance compared with women with high socioeconomic status^44^. This could impact the stage of disease at diagnosis, such that breast cancer in women with low socioeconomic status may therefore be detected in a more advanced stage. Differences in socioeconomic status may also contribute to differences in access to health care including treatment^45^, thereby affecting prognosis. Likewise, low socioeconomic status is associated with a less healthy diet^46^ which may have a negative impact on breast cancer prognosis^47^. Unfortunately, the reported data did not allow for adjustment for socioeconomic status or differences in stage of disease at diagnosis in the analyses.

Additionally, seven out of 13 studies included in this meta-analysis are single-institution studies^24,26,27,29,32–34^. Consequently, to be able to further clarify the influence of overweight on breast cancer prognosis among TNBC patients, there is a need for larger population-based observational studies using validated data. Moreover, BMI is an unrefined measure of body size that does not consider the ratio between adipose tissue and muscle tissue^48^. This could potentially lead to misclassification as individuals classified as normal-weight by BMI, may in fact have metabolic obesity^49^. Future studies are therefore recommended to use more precise measures of body fat when estimating body composition, e.g., dual-energy X-ray absorptiometry scans or waist-hip-ratio^4,50^.

## Conclusion

The results of this, to our knowledge first comprehensive meta-analysis focusing on the role of overweight in TNBC prognosis, highlights the potential negative influence of a modifiable lifestyle factor in a particularly vulnerable patient group already suffering from a worse prognosis due to the aggressiveness of the disease and the lack of targeted treatment possibilities. The possible limitations of the so far limited number of studies, including the possibility of publication bias, indicate a need for additional population-based studies using more precise measures of body fat and enabling the adjustment for differences in possible moderating factors such as disease stage at diagnosis, chemotherapy regimen, and socioeconomic status. The current evidence should also encourage research on the possible efficacy of weight management interventions on TNBC prognosis.

## Methods

The present review was pre-registered with PROSPERO (*reg.no.: CRD42020206102*) and is reported in accordance with the Meta-analysis of Observational Studies in Epidemiology (MOOSE) guidelines^51^.

### Data sources and search strategy

A systematic keyword-based search was conducted in the databases PubMed and Embase. Informed by the *Population Intervention/exposure Comparison Outcome* (PICO) approach^52^, keywords related to *Population* were combined with keywords related to *Exposure* and *Outcome*, e.g., *Triple Negative Breast Cancer AND Prognosis AND Overweight OR Obesity* (the full search string is available in the Supplementary Materials, Table 1). Searches were conducted for the period from the earliest time available until July 7, 2020, together with forward and backward citation tracking (snowballing).

### Selection criteria and data extraction

Study eligibility was established using the PICO approach^52^. Studies were eligible for inclusion if (1) the population included patients diagnosed with TNBC, (2) BMI at diagnosis was included as exposure, (3) the outcome was disease-free survival (DFS [Defined as time from breast cancer diagnosis to first breast cancer event or death]) and/or overall survival (OS [Defined as time from breast cancer diagnosis to death of any cause]), and (4) the study design was longitudinal, investigating the association of overweight with clinical outcome in TNBC patients. We imposed no restrictions on publication year, geographical setting, or length of follow-up. We did not consider case-control studies, studies reporting other effect sizes than hazard ratios, and studies where data on BMI were retrieved >6 months after TNBC diagnosis. Only English language papers in peer-reviewed journals were considered, and “gray literature”, e.g., conference abstracts and dissertations, were not included. Two authors (S.H. and J.O.) independently screened titles and abstracts using the *Covidence systematic review software* (www.covidence.org). After excluding studies based on titles and abstracts, the remaining full text references were reviewed. Disagreements were discussed with a third author (S.B.) until a negotiated conclusion was reached. Data were extracted by one author (S.H.) and coded according to a priori specified characteristics, including study name, patient characteristics, treatment characteristics, exposure (BMI), outcome data (DFS and OS), and risk of bias, and validated by a second author (S.B.).

### Risk of bias assessment

The Newcastle-Ottawa Scale^53^ (NOS) was adapted to assess the risk of bias of the included studies. NOS evaluates the risk of systematic errors in a study design by assessing the following characteristics: (I) Representativeness of the exposed cohort, (II) Selection of the non-exposed cohort, (III) Ascertainment of exposure, (IV) Demonstration that the outcome of interest was not present at start of study, (V) Comparability of cohorts on the basis of the design or analysis, (VI) Assessment of outcome, (VII) Was follow-up long enough for outcomes to occur, and (VIII) Adequacy of follow-up cohorts^53^. Two authors (S.H. and J.O.) independently assessed and scored each study according to the pre-established criteria, and for every present characteristic, one point was dispensed. Disagreements were discussed with a third author (S.B.) until a final score was reached for each study. The risk of bias scores are summarized (Table 4) into a bias judgment^53^.

**Table 4:** Quality assessment based on the Newcastle-Ottawa Scale.

Study	Selection (4)				Comparability^a^ (2)	Outcome (3)			Total score^b^	Risk of bias^c^
	*Representativeness of the exposed cohort*	*Selection of the non-exposed cohort*	*Ascertainment of exposure*	*Demonstration that the outcome of interest was not present at start of study*	*Comparability of cohorts on the basis of the design or analysis*	*Assessment of outcome*	*Was follow-up long enough for outcomes to occur*	*Adequacy of follow-up cohorts*		
*Ademuyiwa (2011)*^47^	★	★	★	☆	★★	★	★	★	8	Low
*Dawood (2012)*^40^	★	★	★	☆	★★	★	★	★	8	Low
*Sparano (2012)*^43^	★	★	★	☆	★☆	★	★	★	7	Low
*Pajares (2013)*^44^	★	★	★	☆	★★	★	★	★	8	Low
*Mowad (2013)*^32^	★	★	★	☆	☆★	★	★	★	7	Low
*Turkoz (2013)*^33^	★	★	★	☆	★☆	★	★	★	7	Low
*Tait (2014)*^37^	★	★	★	☆	☆★	★	★	★	7	Low
*Widschwendter (2015)*^31^	★	★	★	☆	★★	★	★	★	8	Low
*Hao (2015)*^41^	★	★	★	☆	★★	★	★	★	8	Low
*Chen (2016)*^34^	★	★	★	☆	☆☆	★	★	★	6	Moderate
*Bao (2016)*^38^	★	★	★	☆	★★	★	★	★	8	Low
*Al Jarroudi (2017)*^36^	★	★	★	☆	★★	☆	★	☆	6	Moderate
*Wang (2019)*^38^	★	★	★	☆	★☆	★	★	★	7	Low

^a^Stars were given for Comparability if the study adjusted for age and treatment.

^b^Maximum amount of stars for Selection is 4; Maximum amount of stars for Comparability is 2; Maximum amount of stars for Outcome is 3; Maximum amount of stars for Total Score is 9.

^c^A *Total score* of 0–3 indicates high risk, 4–6 a moderate risk, and 7–9 a low risk of bias^24^.

### Overweight and obesity definitions by BMI

Overweight was determined using the World Health Organization (WHO) BMI definition of overweight, i.e., when a patient has a BMI of 25 kg/m^2^ or above the patient is considered overweight, and if the patient has a BMI of less than 25 kg/m^2^ the patient is considered normal-weight^3^. WHO’s BMI definition of overweight differs depending on geographic location; e.g., in Asian populations, the BMI definition for overweight is defined as a BMI of 24 kg/m^2^ or more according to WHO’s Asian-Pacific classification for overweight^41^. WHO further defines subgroups of overweight where patients with a BMI between 25 and 30 kg/m^2^ are considered overweight and patients with a BMI of 30 kg/m^2^ or above are considered obese. In the present study, all patients with a BMI defined as overweight or obese according to WHO are considered overweight and referred to as overweight from this point forward.

### Analytical strategy

Observational cohort studies analyzing data either prospectively or retrospectively were reviewed and subjected to meta-analysis to ascertain the pooled overall effect estimate and its precision. To aid the interpretation of the results, we conducted, as a supplement to the conventional frequentist meta-analysis, a Bayesian Model-Averaged meta-analysis^54^.

### Pooling effect sizes

An inverse variance-weighted random-effects model considering the precision of each study was used in all analyses, with hazard ratios larger than 1.0 taken to indicate an effect in the hypothesized direction, i.e., overweight associated with a shorter DFS or OS. A number of studies reported survival outcomes according to BMI subgroups, e.g., overweight (BMI 25–30) vs normal-weight patients (BMI < 25) and obese (BMI ≥ 30) vs normal-weight patients (BMI < 25). In these cases, we combined the group of overweight (BMI 25–30) and obese patients (BMI ≥ 30) into one group, referred to as overweight patients. This was done to ensure that all patients with a BMI classified as overweight were included in the estimates retrieved from each study. When feasible, additional analyses were conducted for BMI-subgroups of overweight and obese separately (Table 3). The individual and pooled hazard ratios are presented together with the associated 95% confidence intervals in forest plots.

### Heterogeneity

Heterogeneity was investigated using Q and *I*^2^ statistics^55^. Heterogeneity tests aim at determining to which degree the variation in effect sizes reflects true differences (heterogeneity) or sampling error. The *I*^2^ value is an estimate of the between-study variance in a pooled effect estimate that is accounted for by heterogeneity of the effect sizes in the included studies and is assumed to be relatively unaffected by the number of studies^56^. If the results indicated heterogeneity (*I*^2^ > 0.0), we calculated the 95% prediction interval, which estimates the expected range of true effects in 95% of future studies^57^.

### Publication bias

**Fig. 4:**
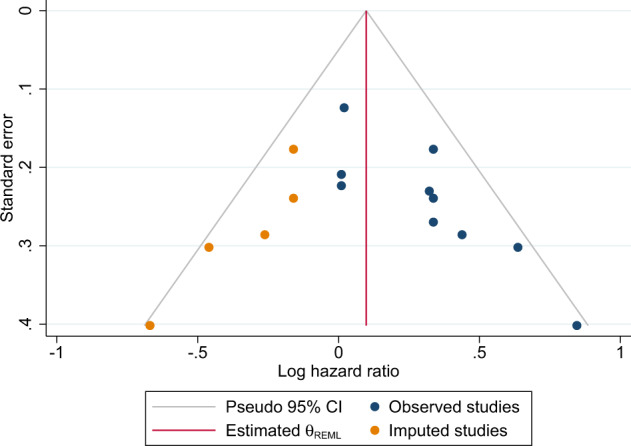
Funnel plot assessing the presence of publication bias in the disease-free survival analyses. * The Duval and Tweedie trim-and-fill method^30^ was used to adjust for publication bias.

**Fig. 5:**
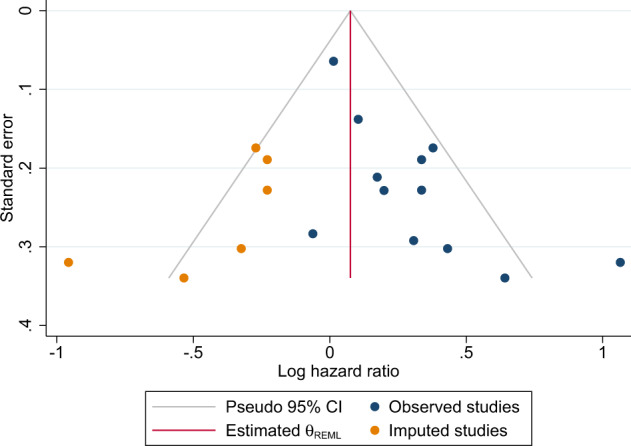
Funnel plot assessing the presence of publication bias in the overall survival analyses. * The Duval and Tweedie trim-and-fill method^30^ was used to adjust for publication bias.

The possibility of publication bias was assessed using funnel plots (Figure 4 and 5) and Egger’s test^58^. If results were suggestive of possible publication bias, sensitivity analyses were conducted by imputing the “missing studies” and calculating adjusted effect estimates using the Duval and Tweedie trim-and-fill method^59^.

### Moderator analyses

To explore possible sources of heterogeneity (*I*^2^ > 0.0), we examined, with meta-regression based on random-effects models and estimated with maximum likelihood method, the role on the effect size of two possible moderators, i.e., median follow-up time in months and the BMI used as cutoff for overweight in each study.

### Bayesian analysis

A supplementary Bayesian Model-Averaged meta-analysis^54^ of the effects of overweight on TNBC prognosis examined the results of four models: (a) fixed-effect null hypothesis (fH_0_), (b) fixed-effect alternative hypothesis (fH_1_), (c) random-effects null hypothesis (rH_0_), and (d) random effects alternative hypothesis (rH_1_). Bayesian Model-Averaged analysis thus avoids selecting either a fixed- or random-effects model and addresses two questions in light of the observed data: What is the plausibility that the overall effect is non-zero and is there between-study variability in the effect size? We chose an uninformed prior probability, i.e., 25%, of each of the four models and 2,000 iterations. Concerning parameter distributions, we chose previously recommended defaults^54^. We thus used a zero-centered Cauchy prior with a scale of 0.707 for the effect size. To have zero indicating the null effect, the hazard ratios and the upper and lower limits were log-transformed. For the between-study variation, we used an empirically informed prior distribution of non-zero between-study deviation estimates based on effect sizes from 705 meta-analyses published in Psychological Bulletin between 1990 and 2013^60^. This distribution has been approximated by an Inverse-Gamma (1, 0.15) prior on the standard deviation (Tau)^54^.

The frequentist analyses were performed using Comprehensive Meta-Analysis, version 3^61^. The supplementary Bayesian analyses were conducted with JASP, Version 0.12.2^62^.

## Supplementary information

Supplementary Information

## Data Availability

The data underlying this article are available in the article and in its online supplementary material.

## Code availability

The code developed during this study is available upon reasonable request. Analyses were performed using Comprehensive Meta-Analysis, version 3^61^ and JASP, Version 0.12.2^62^. Figures were made using Stata®, version 16 (StataCorp, College Station, Texas, USA).

## References

[1] Bray F (2018). Global cancer statistics 2018: GLOBOCAN estimates of incidence and mortality worldwide for 36 cancers in 185 countries. Ca. Cancer J. Clin..

[2] Meldrum DR, Morris MA, Gambone JC (2017). Obesity pandemic: causes, consequences, and solutions—but do we have the will?. Fertil. Steril..

[3] Obesity and overweight. https://www.who.int/news-room/fact-sheets/detail/obesity-and-overweighthttps://www.who.int/news-room/fact-sheets/detail/obesity-and-overweight (2021).

[4] Iyengar NM (2019). Association of body fat and risk of breast cancer in postmenopausal women with normal body mass index: a secondary analysis of a randomized clinical trial and observational study. JAMA Oncol..

[5] Ewertz M (2011). Effect of obesity on prognosis after early-stage breast cancer. J. Am. Soc. Clin. Oncol..

[6] Chan DSM (2014). Body mass index and survival in women with breast cancer-systematic literature review and meta-analysis of 82 follow-up studies. Ann. Oncol. J. Eur. Soc. Med. Oncol..

[7] Nuttall FQ (2015). Body mass index: obesity, bmi, and health: a critical review. Nutr. Today.

[8] Sommer I (2020). The performance of anthropometric tools to determine obesity: a systematic review and meta-analysis. Sci. Rep..

[9] Howlader N., N A. SEER Cancer Statistics Review, 1975–2016, National Cancer Institute. Bethesda, MD, (2019).

[10] Pierobon M, Frankenfeld CL (2013). Obesity as a risk factor for triple-negative breast cancers: a systematic review and meta-analysis. Breast Cancer Res. Treat..

[11] Gershuni V (2017). Breast cancer subtype distribution is different in normal weight, overweight, and obese women. Breast Cancer Res. Treat..

[12] Apostolopoulos V (2016). The complex immunological and inflammatory network of adipose tissue in obesity. Mol. Nutr. Food Res..

[13] Kim C-S (2006). Circulating levels of MCP-1 and IL-8 are elevated in human obese subjects and associated with obesity-related parameters. Int. J. Obes..

[14] Franklin RA (2014). The cellular and molecular origin of tumor-associated macrophages. Science.

[15] Noy R, Pollard JW (2014). Tumor-associated macrophages: from mechanisms to therapy. Immunity.

[16] Yang H (2014). Toll-like receptor 4 prompts human breast cancer cells invasiveness via lipopolysaccharide stimulation and is overexpressed in patients with lymph node metastasis. PLoS ONE.

[17] Rinkenbaugh AL, Baldwin AS (2016). The NF-κB pathway and cancer stem. Cells.

[18] Dent R (2007). Triple-negative breast cancer: clinical features and patterns of recurrence. Clin. Cancer Res..

[19] Cornelia Liedtke (2008). Response to neoadjuvant therapy and long-term survival in patients with triple-negative breast cancer. J. Clin. Oncol..

[20] Bianchini G, Balko JM, Mayer IA, Sanders ME, Gianni L (2016). Triple-negative breast cancer: challenges and opportunities of a heterogeneous disease. Nat. Rev. Clin. Oncol..

[21] Mei, L. et al. Association between obesity with disease-free survival and overall survival in triple-negative breast cancer. *Medicine (Baltimore)* **97**, (2018). 10.1097/MD.0000000000010719 PMC5959383 29742734

[22] Widschwendter P (2015). The influence of obesity on survival in early, high-risk breast cancer: results from the randomized SUCCESS A trial. Breast Cancer Res.

[23] Mowad R (2013). Does obesity have an effect on outcomes in triple-negative breast cancer?. J. Surg. Res..

[24] Chen H, Ding A, Wang M (2016). Impact of central obesity on prognostic outcome of triple negative breast cancer in Chinese women. SpringerPlus.

[25] Wang K (2019). Clinicopathologic and prognostic significance of body mass index (BMI) among breast cancer patients in Western China: a retrospective multicenter cohort based on Western China Clinical Cooperation Group (WCCCG). Biomed. Res Int.

[26] Al Jarroudi O, Abda N, Seddik Y, Brahmi SA, Afqir S (2017). Overweight: is it a prognostic factor in women with triple-negative breast cancer?. Asian Pac. J. Cancer Prev..

[27] Tait S (2014). Body mass index, diabetes, and triple-negative breast cancer prognosis. Breast Cancer Res. Treat..

[28] Bao P-P (2016). Body mass index and weight change in relation to triple-negative breast cancer survival. Cancer Causes Control.

[29] Turkoz FP (2013). The prognostic impact of obesity on molecular subtypes of breast cancer in premenopausal women. J. BUON . J. Balk. Union Oncol..

[30] Sparano JA (2012). Obesity at diagnosis is associated with inferior outcomes in hormone receptor-positive operable breast cancer. Cancer.

[31] Pajares B (2013). Obesity and survival in operable breast cancer patients treated with adjuvant anthracyclines and taxanes according to pathological subtypes: a pooled analysis. Breast Cancer Res..

[32] Dawood S (2012). Impact of body mass index on survival outcome among women with early stage triple-negative breast cancer. Clin. Breast Cancer.

[33] Hao S (2015). Overweight as a prognostic factor for triple-negative breast cancers in Chinese Women. PLoS ONE.

[34] Ademuyiwa FO (2011). Impact of body mass index on clinical outcomes in triple-negative breast cancer. Cancer.

[35] Goodman SN (1999). Toward evidence-based medical statistics. 2: the Bayes factor. Ann. Intern Med..

[36] Kaestner SA, Sewell GJ (2007). Chemotherapy dosing part I: scientific basis for current practice and use of body surface area. Clin. Oncol. R. Coll. Radio..

[37] Si S (2020). Body surface area, height, and body fat percentage as more sensitive risk factors of cancer and cardiovascular disease. Cancer Med..

[38] Redlarski G, Palkowski A, Krawczuk M (2016). Body surface area formulae: an alarming ambiguity. Sci. Rep..

[39] Griggs JJ, Sorbero MES, Lyman GH (2005). Undertreatment of obese women receiving breast cancer chemotherapy. Arch. Intern. Med..

[40] Lambert J (2011). Statistics in brief: how to assess bias in clinical studies?. Clin. Orthop..

[41] Inoue, S. *et al*. The Asia-Pacific perspective: redefining obesity and its treatment. *Syd. Health Commun. Aust. Pty Ltd* (2000).

[42] Ballinger TJ, Jiang G, Kassem N, Radovich M, Schneider BP (2021). Impact of body mass index on presence of ctDNA and disease recurrence after neoadjuvant chemotherapy for triple-negative breast cancer: analysis from BRE12-158. Clin. Cancer Res. J. Am. Assoc. Cancer Res..

[43] Lohmann, A. E. et al. Association of obesity with breast cancer outcome in relation to cancer subtypes: a meta-analysis. *JNCI J. Natl. Cancer Inst*. (2021) 10.1093/jnci/djab023. 10.1093/jnci/djab023 PMC8562970 33620467

[44] Lundqvist A, Andersson E, Ahlberg I, Nilbert M, Gerdtham U (2016). Socioeconomic inequalities in breast cancer incidence and mortality in Europe-a systematic review and meta-analysis. Eur. J. Public Health.

[45] Bradley CJ, Given CW, Roberts C (2002). Race, Socioeconomic Status, and Breast Cancer Treatment and Survival. JNCI J. Natl Cancer Inst..

[46] French SA, Tangney CC, Crane MM, Wang Y, Appelhans BM (2019). Nutrition quality of food purchases varies by household income: the SHoPPER study. BMC Public Health.

[47] Zheng J (2018). Association between post-cancer diagnosis dietary inflammatory potential and mortality among invasive breast cancer survivors in the Women’s health initiative. Cancer Epidemiol. Prev. Biomark..

[48] Okorodudu DO (2010). Diagnostic performance of body mass index to identify obesity as defined by body adiposity: a systematic review and meta-analysis. Int. J. Obes..

[49] Gómez-Ambrosi J (2012). Body mass index classification misses subjects with increased cardiometabolic risk factors related to elevated adiposity. Int. J. Obes..

[50] Swainson MG, Batterham AM, Tsakirides C, Rutherford ZH, Hind K (2017). Prediction of whole-body fat percentage and visceral adipose tissue mass from five anthropometric variables. PLoS ONE.

[51] Meta-analysis of observational studies in epidemiology: a proposal for reporting. Meta-analysis Of Observational Studies in Epidemiology (MOOSE) group | The EQUATOR Network. https://www.equator-network.org/reporting-guidelines/meta-analysis-of-observational-studies-in-epidemiology-a-proposal-for-reporting-meta-analysis-of-observational-studies-in-epidemiology-moose-group/. 10.1001/jama.283.15.2008 10789670

[52] Henderson, A. R. Evidence-Based Medicine—How to Practice and Teach EBM. D. L. Sackett, W. S. Richardson, W. Rosenberg, and R. B. Haynes. New York: Churchill Livingstone, 1997, 250 pp. Paperback, $24.99. ISBN 0-443-05686-2. *Clin. Chem*. **43**, 2014–2014 (1997).

[53] Wells, G. et al. The Newcastle-Ottawa Scale (NOS) for Assessing the Quality of Nonrandomised Studies in Meta-Analyses. in (2014).

[54] Gronau QF (2017). A Bayesian model-averaged meta-analysis of the power pose effect with informed and default priors: the case of felt power. Compr. Results Soc. Psychol..

[55] Higgins J. P. T., J. Thomas Cochrane Handbook for Systematic Reviews of Interventions version 6.1 (updated September 2020). (2020).

[56] Higgins JPT, Thompson SG, Deeks JJ, Altman DG (2003). Measuring inconsistency in meta-analyses. BMJ.

[57] Plea for routinely presenting prediction intervals in meta-analysis | BMJ Open. https://bmjopen.bmj.com/content/6/7/e010247. 10.1136/bmjopen-2015-010247 PMC4947751 27406637

[58] Egger M, Smith GD, Schneider M, Minder C (1997). Bias in meta-analysis detected by a simple, graphical test. BMJ.

[59] Duval S, Tweedie R (2000). Trim and fill: a simple funnel-plot-based method of testing and adjusting for publication bias in meta-analysis. Biometrics.

[60] van Erp, S., Verhagen, A. J., Grasman, R. P. P. P. & Wagenmakers, E.-J. Estimates of between-study heterogeneity for 705 meta-analyses reported in Psychological Bulletin from 1990–2013. *J. Open Psychol. Data* (2017).

[61] Borenstein, M., Hedges, L., Higgins, J. & Rothstein, H. *Comprehensive Meta-Analysis Version 3*. (Biostat, 2013).

[62] JASP Team. *JASP*. (2020).

