# Supplementary material for: Overweight and prognosis in triple-negative breast cancer patients: a systematic review and meta-analysis
Source: NPJ Breast Cancer. 2021 Sep 10;7:119. doi: 10.1038/s41523-021-00325-6 (PMC8433348; doi:10.1038/s41523-021-00325-6)
Supplement: Supplementary file 1 — Supplementary Information [file 41523_2021_325_MOESM1_ESM.pdf]

Supplementary material

Supplementary tables

Supplementary Table 1. *Search protocol*

**Research Question**	**Databases, Resources, and limits**		
	**Databases**	**Grey Literature**	**Limits**
Does clinical outcome following triple-negative breast cancer differ between overweight and non-overweight patients?	MEDLINE (PubMed) & Embase +Reference List Searching (Snowballing) and citation search.	Not searched	Language: English, Years: All, Age Groups: ≥ 18 years, Publiation Types: Peer-review Geographical: All
**Databases searched:**			
**Search 1:**			
Type of search	Initial screening of existing systematical reviews in PubMed		
Search string	(breast cancer) AND (survival OR prognosis OR recurrence*) AND (obesity OR overweight OR BMI) and meta-analysis		
**Yielded references: 75**	**Duplicates removed: N/A**	**Total added: N/A**	**Date conducted: 01.07.2020**
**Search 2:**			
Type of search	Final saturated MesSH-search in PubMed		
Search string	("Triple Negative Breast Neoplasms"[MeSH]) AND "Overweight"[MeSH] OR “Obesity” [MeSH])		
**Yielded references: 31**	**Duplicates removed: 0**	**Total added: 31**	**Date conducted: 07.07.2020**
**Search 3:**			
Type of search	Initial screening of Embase		
Search string	('triple negative breast cancer'/exp OR 'triple negative breast cancer') AND ('prognosis'/exp OR prognosis) AND ('obesity'/exp OR obesity)		
**Yielded references: 163**	**Duplicates removed: N/A**	**Total added: N/A**	**Date conducted: 01.07.2020**
**Search 4:**			
Type of search	Final saturated search in Embase		
Search string	‘triple negative breast cancer’ AND overweight AND prognosis		
**Yielded references: 47**	**Duplicates removed: 10**	**Total added: 37**	**Date conducted: 07.07.2020**
**Search 5:**			
Type of search	Reference lists		
**Yielded references: 5**	**Duplicates removed: N/A**	**Total added: 5**	**Date conducted: 07.07.2020**

*N/A = Not applicable;

**Protocol modified from *http://guides.library.stonybrook.edu/c.php?g=226681&p=1502561#s-lg-box-wrapper-5374018* 01.07.2020

Supplementary Table 2. *Event definition of disease-free and overall survival in the included studies*

**Study**	**Event definition DFS**	**Event definition OS**
Dawood (2012)[42]	N/R	Death
Mowad (2013)[34]	Date of diagnosis to first recurrence (local or distant)	Death from any cause
Turkoz (2013)[49]	The interval from date of diagnosis to the date of locoregional or distant recurrence	Date of diagnosis to the date of death from breast cancer
Tait (2014)[39]	Time from surgery to any invasive breast recurrence (local or distant) or death	Time from diagnosis to death from any cause
Widschwendter (2015)[33]	Local, contralateral and distant disease recurrence, secondary primary tumors and death	Death from any cause
Hao (2015)[43]	N/R	Death from any cause
Chen (2016)[36]	Diagnosis to first recurrence/metastasis	Death from any cause
Bao (2016)[40]	Cancer recurrence/metastasis or death related to breast cancer	Death from any cause
Al Jarroudi (2017)[38]	Event not defined	Event not defined
Liu (2018)[41]	N/R	Death
Wang (2019)[37]	Date of diagnosis to the locoregional or distant recurrence or death from any cause	N/R

**DFS* = Disease-free survival; *OS* = Overall survival; *N/R* = Not reported

Supplementary figures

Supplementary Figure 1. *Meta-analysis of observational studies comparing recurrence-free survival in normal-weight and overweight.*

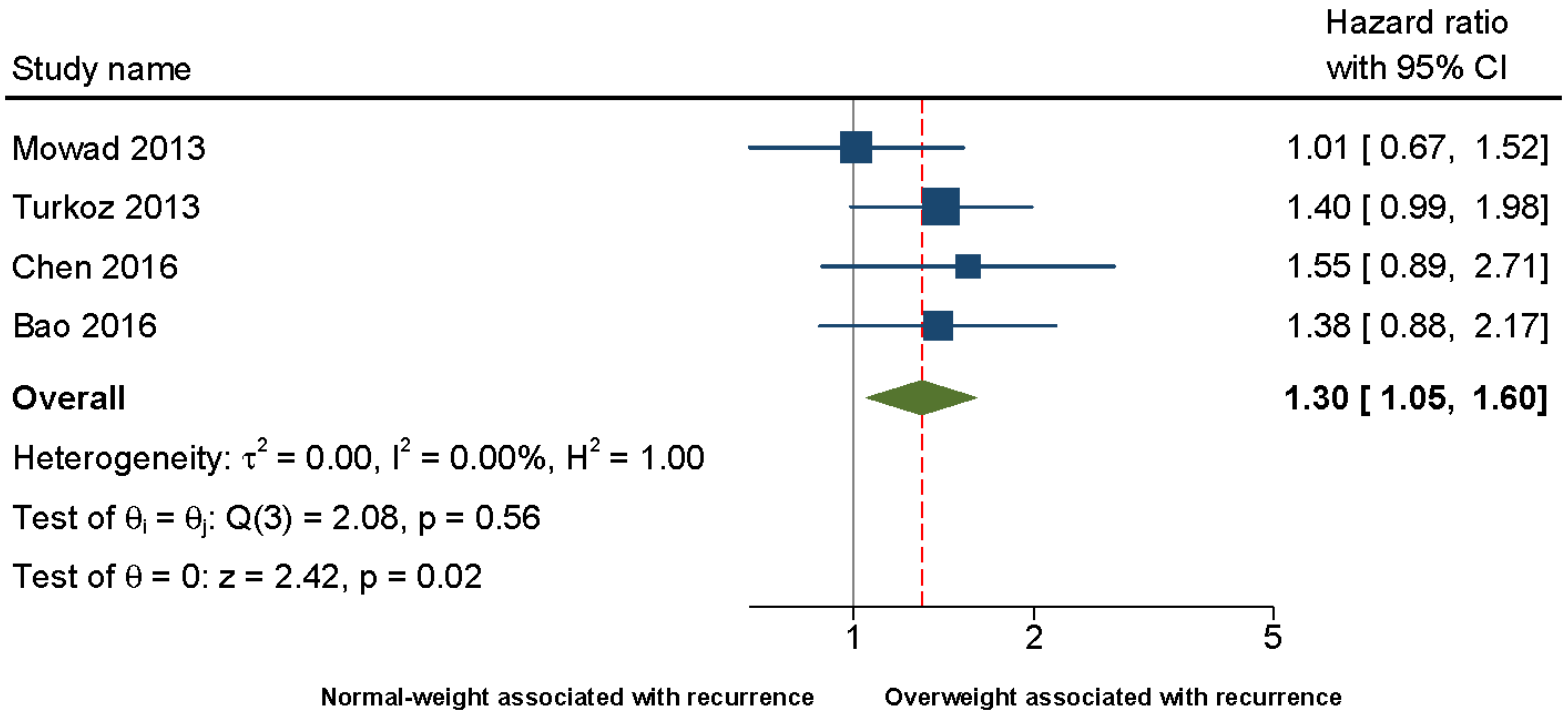

**Recurrence-free survival was defined as the time from breast cancer diagnosis to first recurrence.*
